# Quality of Life with Late-Onset Pompe Disease: Qualitative Interviews and General Public Utility Estimation in the United Kingdom

**DOI:** 10.36469/001c.68157

**Published:** 2023-03-03

**Authors:** Lena Hubig, Anna-Katrine Sussex, Alasdair MacCulloch, Derralynn Hughes, Ryan Graham, Liz Morris, Syed Raza, Andrew J. Lloyd, Amanda Sowinski, Katy Gallop

**Affiliations:** 1 Acaster Lloyd Consulting Ltd, London, UK; 2 Amicus Therapeutics UK Ltd, Marlow, UK; 3 Lysosomal Storage Disorders Unit, Royal Free London NHS Foundation Trust and University College London, UK; 4 GrahamAmicus Therapeutics UK Ltd, Marlow, UK; 5 Addenbrooke’s Hospital, Cambridge, UK; 6 Amicus Therapeutics UK Ltd, Marlow

**Keywords:** Pompe disease, glycogen storage disease type II, health-related quality of life, EQ-5D-5L, time trade-off, utility

## Abstract

**Background:** Late-onset Pompe disease (LOPD) is a rare, progressive neuromuscular condition typically characterized by weakness of skeletal muscles, including those involved in respiration and diaphragmatic dysfunction. Individuals with LOPD typically eventually require mobility and/or ventilatory support. **Objectives:** This study aimed to develop health state vignettes and estimate health state utility values for LOPD in the United Kingdom. **Methods:** Vignettes were developed for 7 health states of LOPD with states defined in terms of mobility and/or ventilatory support. Vignettes were drafted based on patient-reported outcome data from the Phase 3 PROPEL trial (NCT03729362) and supplemented by a literature review. Qualitative interviews with individuals living with LOPD and clinical experts were conducted to explore the health-related quality-of-life (HRQoL) impact of LOPD and to review the draft vignettes. Vignettes were finalized following a second round of interviews with individuals living with LOPD and used in health state valuation exercises with people of the UK population. Participants rated the health states using the EQ-5D-5L, visual analogue scale, and time trade-off interviews. **Results:** Twelve individuals living with LOPD and 2 clinical experts were interviewed. Following the interviews, 4 new statements were added regarding dependence on others, bladder control problems, balance issues/fear of falling, and frustration. One hundred interviews with a representative UK population sample were completed. Mean time trade-off utilities ranged from 0.754 (SD = 0.31) (no support) to 0.132 (SD = 0.50) (invasive ventilatory and mobility support–dependent). Similarly, EQ-5D-5L utilities ranged from 0.608 (SD = 0.12) to -0.078 (SD = 0.22). **Discussion:** The utilities obtained in the study are consistent with utilities reported in the literature (0.670-0.853 for nonsupport state). The vignette content was based on robust quantitative and qualitative evidence and captured the main HRQoL impacts of LOPD. The general public rated the health states consistently lower with increasing disease progression. There was greater uncertainty around utility estimates for the severe states, suggesting that participants found it harder to rate them. **Conclusion:** This study provides utility estimates for LOPD that can be used in economic modeling of treatments for LOPD. Our findings highlight the high disease burden of LOPD and reinforce the societal value of slowing disease progression.

## INTRODUCTION

Pompe disease is a rare, recessive neuromuscular disorder with a population incidence of 1:20 000 to 1:30 000, depending on geographic region, ethnicity, and type of diagnosis.^1,2^ Pompe disease is caused by a mutation in the gene responsible for the production of acid α-glucosidase, an enzyme which breaks down lysosomal glycogen. Individuals with this gene mutation are deficient of this enzyme, and the overaccumulation of lysosomal glycogen leads to progressive disruption of cellular function, in particular in the heart (in infants), skeletal muscles, and diaphragm.^3^ Infantile-onset Pompe disease is characterized by the presentation of symptoms within the first 4 months of life, while individuals living with late-onset Pompe disease (LOPD) can begin experiencing symptoms from childhood through to adulthood. Severity and age at onset depend on the level of deficiency, with most patients having LOPD.^4^

Most patients with LOPD experience slow and progressive loss of muscle function, typically starting with the trunk and lower limbs and deterioration of respiratory muscles.^5,6^ Over time, this progressive loss of muscle function may lead to the need for mobility and ventilatory support. The fatigue and muscle weakness experienced by individuals living with LOPD impacts their social and professional health-related quality of life (HRQoL) by reducing their participatory abilities through need to rest,^7,8^ which also has significant emotional impact.^9,10^ Untreated, there is a significant impact on mortality and morbidity. In one observational study, in the absence of treatment, the median age of death from LOPD was 56 years,^11^ with respiratory failure accounting for more than 70% of deaths.^6,12^

Enzyme replacement therapy (ERT) with alglucosidase alfa was the first pharmacological treatment for LOPD that aimed to slow disease progression, but the effectiveness was limited in some people.^3^ In recent years, novel approaches to ERT, including next-generation ERT and a 2-component approach with a novel ERT plus enzyme stabilizer, have been evaluated.^13^ New treatments will typically go through a process of health technology assessment to support decision-making. In many countries, this will include an assessment of cost-effectiveness of the treatment.^10^ Outcomes expressed in terms of quality-adjusted life-years will require quality of life weights or utilities; in LOPD, such data are limited.^11^

The objective of this study was to estimate UK societal utility weights for different states of LOPD. Health state vignettes were drafted and validated based on patient-reported outcome (PRO) data from a clinical trial in LOPD, a targeted literature review, and in-depth qualitative interviews with individuals living with LOPD and healthcare professionals (HCP) with experience caring for individuals living with LOPD. The vignettes were then valued by members of the UK general public to obtain utility values using the EQ-5D-5L and time trade-off (TTO) valuation methods.

## METHODS

### Study Design

Seven adult health state vignettes (Table 1) were developed, which were defined in terms of level of mobility and ventilatory support required. The vignettes were constructed using data from different independent sources, including a clinical trial, a targeted literature review, and 2 rounds of qualitative interviews with people living with LOPD and HCPs experienced in LOPD. The final vignettes were valued in interviews with members of the UK general population using a visual analog scale (VAS), TTO method, and the EQ-5D-5L.

**Table 1.** Health States Defined by Assistive Technology Dependence

**Health State**		**Mobility Support**	**Ventilatory Support**
1	No support	None	None
2	Intermittent mobility support	Use of mobility support (walking aid, wheelchair, or motorized scooter) some of the time	None
3	Intermittent ventilatory support	None	Use of ventilatory support during the night or sometimes during the day
4	Intermittent ventilatory and mobility support	Use of mobility support (walking aid, wheelchair, or motorized scooter) some of the time	Use of ventilatory support during the night or sometimes during the day
5	Mobility support-dependent	Use of wheelchair or motorized scooter all of the time	None
6	Mobility support-dependent and intermittent ventilatory support	Use of wheelchair or motorized scooter all of the time	Use of ventilatory support during the night or sometimes during the day
7	Mobility support-dependent and invasive ventilatory support-dependent	Use of wheelchair or motorized scooter all of the time	Use of invasive ventilatory support

The Western Institutional Review Board (WIRB; March 2022) reviewed the study protocol and declared the study minimal risk and therefore exempt from ethical review.

### Clinical Trial Data Review

The Phase 3 PROPEL clinical trial (NCT03729362)^14^ collected subjects’ self-reported HRQoL using a PRO measure called the Rasch-built Pompe-specific activity questionnaire (R-PAct)^15^ and the EQ-5D-5L. PROPEL included only individuals living with LOPD who were ambulatory; data from this study supported the ambulatory health state vignettes (no support, intermittent mobility/ventilatory support). Nonambulatory health states 5, 6, and 7 were extrapolated from the existing health states and adapted to mobility support dependence and invasive ventilation dependence as appropriate.

The R-PAct consists of 18 items and was developed to measure the limitation of functioning and activities in individuals living with Pompe disease.^15^ The R-PAct was validated in individuals living with mild to very severe Pompe disease and was shown to accurately measure the limitations individuals living with Pompe experience.^15^ It has since been used to measure progression of LOPD and to evaluate the effect of ERT in LOPD.^16–18^ The EQ-5D-5L is a measure of HRQoL that asks participants to rate their health in terms of 5 dimensions: mobility, self-care, usual activities, pain/discomfort, and anxiety/depression.^19^

Draft health state vignettes were designed based on all EQ-5D-5L dimensions and a subset of R-PAct items that described direct HRQoL impacts and were not covered by other items, such as walking short distances, walking up and down stairs, standing from a seated position, getting dressed, preparing a meal, and eating.

Trial subjects were classified into the model health states (Table 1) based on their documented use of mobility and ventilatory support. Counts (%) of item-level responses were calculated for all subjects classified into each health state. For each EQ-5D-5L and R-PAct item, the most frequently reported level was selected to describe the functioning of and disease impact on individuals living with Pompe disease in each health state. If the responses were bimodal, either the median response or a gradient (eg, slight to moderate problems) was used. If there were few subjects classified into a health state, data from earlier states informed the description based on the assumption that functioning does not improve with disease progression.

### Literature Review

A literature review was conducted to support the health state vignettes drafted based on the PRO data, provide evidence for the description of mobility support dependence and invasive ventilation, and identify further HRQoL dimensions not captured in the R-PAct or EQ-5D-5L.

Fourteen studies were identified in a targeted literature review of symptoms and impacts of LOPD on people’s life and functioning.^20^ A further 41 testimonies, unsolicited by the study, of individuals living with LOPD or their caregivers, were identified from the International Pompe Association website and reviewed. The testimonies were written to broaden awareness of Pompe disease and differences in impacts on individuals. Descriptions about symptoms, impact on functioning and daily activities, and experience with assistive technologies were extracted and summarized in a literature extraction grid.

### Qualitative Interviews With Healthcare Professionals and Individuals Living With LOPD

Individuals with a self-reported diagnosis of LOPD over the age of 18 and able to read and speak English fluently and provide their informed consent to participate were recruited from the Pompe disease community via patient association groups, Pompe Support Network UK and the Association for Glycogen Storage Disease UK. Eligible participants were invited to take part in a 1-hour interview (over Zoom). Interviews were conducted by experienced interviewers using a semistructured interview guide. In the interview, participants were first asked to describe their experience with LOPD and its impact on different aspects of their HRQoL. The second part of the interview used the think-aloud approach for cognitive debriefing followed by probe questions about different aspects of the vignettes.^21^ Participants reviewed the draft vignettes for their own current and previously experienced health states and provided feedback on the vignettes’ validity and accuracy. The vignettes were revised iteratively and finalized following interviews with HCPs and a second round of interview reviews by individuals living with LOPD. The final health state vignettes are provided in the **Supplementary Material, Appendix 1**.

### Health State Utility Evaluation

For health state utility evaluation interviews, 100 residents of the United Kingdom at least 18 years old, representative of most recent census data,^22^ were recruited using convenience and snowball sampling. Participants who were carers for children with serious chronic conditions were excluded.

Participants were given information about the study and completed consent and background sociodemographic forms prior to the interviews. In a 1-hour online (Zoom) interview, participants rated their own current HRQoL using the EQ-5D-5L. Participants then valued each health state using a VAS, the EQ-5D-5L, and the TTO interview method. For the interviews, the names of the health states were removed and the states were randomized into 4 order sets to which participants were equally assigned. All interviews were conducted by experienced TTO interviewers.

At the start of the valuation, participants rated the health states on a VAS from 0 (worst health possible) to 100 (full health). Participants first rated a “dead” state using the VAS scale, to aid calibration of their health state valuations. Participants then read through each health state in turn and imagined themselves in the described health state and rated each state on the VAS.

**Figure 1.**
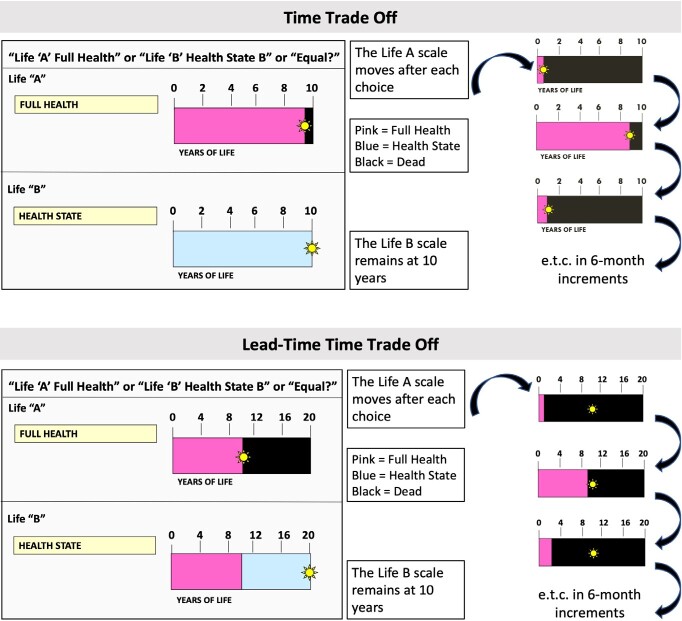
Diagram of Time-Trade-off and Lead-Time Time Trade-off Methods

The interview then moved on to the TTO method, a recognized interviewing technique for generating utility valuations of health states.^23^ For each health state, the participant read the vignette carefully, imagining they were in the health state described. They were then asked to choose between remaining in the health state without improvement for 10 years (followed by death), or to live a shorter number of years in “full health” followed by death. The process incorporates a “ping-pong” approach with participants trading months of full health (in 6-month increments) to avoid living in the health state until their trade-offs iteratively narrow to the point of indifference, where the participant believes the two prospects are the same (Figure 1).

If participants reported a preference for 0 years of full health (ie, immediate death) over 10 years in the described health state, a lead-time TTO method was used, in which each health state was preceded by 10 years of full health (Figure 1). By increasing the number of years that can be traded, the lead-time method allows participants to indicate how much worse than dead they believe a health state to be.

Finally, participants rated each health state using the EQ-5D-5L. In this part of the valuation, participants were asked to continue imagining themselves in the state of health described and to answer the EQ-5D-5L questions for each health state.

Participants’ ratings were assessed for consistency, and participants and interviewers confirmed that participants understood the exercise and were engaged.

The EQ-5D-5L rating for each state was scored using the National Institute for Health and Care Excellence (NICE) Decision Support Unit UK mapping function for the EQ-5D-5L.^24,25^ TTO data were scored according to the point of indifference. The VAS ratings for each vignette were rescaled such that the value for the dead state was fixed at 0 and all other values varied between 100 and the worst health state. All ratings were summarized descriptively, and the mean, SD, and 95% confidence interval were calculated for each health state separately.

The analysis was done using R 4.1.2^26^ and the eq5d R package.^25^ Participants’ background characteristics were summarized descriptively (continuous variables: mean, SD; categorical variables: count, %).

## RESULTS

### Vignette Development

The first vignette draft was developed based on the baseline item–level response data of 87 participants of the Phase 3 PROPEL clinical trial who completed the EQ-5D-5L and 71 PROPEL participants who completed the R-PAct. **Appendix 2** provides detailed results of the trial data review. On the EQ-5D-5L, participants with documented intermittent ventilatory support reported more problems than participants with no documented support or documented intermittent mobility support. On the R-PAct, most participants reported difficulties or being unable to walk more than 1 km, and most reported being unable to run and practice any sport. Compared with participants with documented intermittent ventilatory support, those without had fewer difficulties with usual activities or self-care.

Only 1 trial participant had documented use of intermittent ventilatory and mobility support. If that individual reported better functioning than subjects with documented intermittent mobility support, the descriptions of the earlier health state were carried forward due to the assumption that the disease progresses.

The targeted literature review suggested that mobility is impaired through muscle weakness,^27,28^ low endurance (breathing difficulties),^29^ and fatigue.^30^ Individuals living with LOPD avoid using assistive technology as it was perceived as losing the battle against the disease,^31^ although assistive technology, particularly wheelchairs, can also reinstate freedom.^31–33^ They often use different types of mobility support and start using a wheelchair when leaving their home.^9,31–33^ Wheelchair use is associated with lower physical and social functioning.^34^ Individuals living with LOPD first start using ventilatory support during the night or when sleeping, and then progress to daytime use also.^35^ The main benefit of nocturnal ventilatory support was described as improved sleep, reduced fatigue, and improved general well-being.^29,30,33^ Other impacts described in the literature concerned consequences on social life and relationships due to reduced outings and increased social isolation^9,36^ and dependence on caregiving by family members.^33,37^

The literature review identified additional impacts on sleep, fatigue, social functioning, and relationships not captured in the PROs, which was added to the health state vignettes. The findings also provided support for the descriptions of health states of individuals with LOPD who were not included in the clinical trial.

### Qualitative Interviews With Healthcare Professionals and Individuals With LOPD

Twelve adults with LOPD were interviewed, of which 4 were interviewed twice. Eleven participants provided information about their symptoms, impacts experienced, and assistive technology use. Of those, 8 also reviewed draft vignettes. One participant only provided feedback on draft vignettes. Participants were on average 62 (SD = 9) years old, and 58% (n = 7) were female. Participants were diagnosed with LOPD on average 12 (SD = 9) years ago, and 75% were receiving treatment for LOPD (n = 9). Intermittent mobility aid users typically used different mobility aids indoors and outdoors depending on the length of time expected to be spent outside. Participants dependent on mobility aids used a wheelchair both inside and outside their home, mainly to reduce risk of falling. Intermittent ventilatory support users were dependent on noninvasive ventilation when lying down or sleeping. Some participants also used it the day following particular tiredness or exertion.

Two UK-based HCPs were interviewed who each had approximately 20 years of experience caring for individuals living with LOPD.

### Symptom Burden and HRQoL Impact

**Interviews with individuals living with LOPD.** Most interview participants living with LOPD reported breathing-related sleep disturbance, and some using nighttime noninvasive ventilation reported breathlessness. All individuals living with LOPD reported fatigue and muscle weakness, and most also reported pain. Some linked this to muscle weakness and subsequent muscle overuse; for others, pain was triggered by usual activities such as standing, bending over, and dressing.

Most participants experienced falls, which they attributed to either muscle weakness or balance problems in which they are unable to restabilize themselves following a stumble. Fear of falling was cited by some participants as influencing them to begin full-time wheelchair use. Some reported that due to leg muscle weakness, they used their upper bodies to rise from a chair and to support them when climbing stairs. The majority of wheelchair-dependent users only could stand from seated using an elevating feature of their wheelchair. Several participants reported bladder retention problems, needing to use the toilet frequently and urgently. The impact of this was worsened by their slow movement due to muscle weakness and imbalance.

Many participants reported difficulties washing or dressing, particularly their lower bodies, due to challenges standing unsupported or bending over due to muscle weakness. People’s ability to do housework with LOPD was similarly impacted. Some individuals living with LOPD had difficulties carrying things due to arm muscle weakness or because they need one hand for stability or using a mobility aid when picking things up or walking. Others described being only able to do chores at waist height due to difficulties holding objects above that height or bending over because of muscle weakness.

Most people interviewed found LOPD impacted their working lives. Some reduced working hours and others retired early, citing balance problems, pain, and a reduced ability to travel.

Half of participants reported significant dependence or reliance on others, 3 of whom had full time carers, and 4 of whom reported not leaving their home alone. Over half of participants gave up their hobbies as they were no longer safely able to take part due to their LOPD. Others could take part only in sedentary social activities, such as having dinner, and thus miss out on other leisure and social activities.

Other individuals living with LOPD reported avoiding social activities due to fear of falling and urinary urgency, which restricts them to activities with nearby accessible toilets. When leaving their home, they must plan ahead, ensuring that the destination has limited tripping hazards, toilets nearby, and chairs and toilets sufficiently raised to allow them to get out of them without assistance. Anxiety about these requirements caused some participants to leave home less often. Individuals with LOPD also reported impacts on their romantic relationships as well as relationships with friends and family. Some found that doing fewer activities with friends led to reduced social contact, while others felt supported by friends. Similarly, some individuals reported having become closer to their family due to LOPD, while others described awkwardness and conflict. Two participants also divorced or broke off an engagement due to lack of support from their romantic partner related to their LOPD.

LOPD and its impacts on people’s daily activities were also described as emotionally impactful. Most individuals living with LOPD were frustrated by their condition, with some focusing on tasks they could no longer do. Some individuals experienced depression, and all reported anxiety. Fear of falling was especially common.

**Interviews with healthcare professionals.** The HCPs described that while people living with LOPD can initially present with either respiratory or mobility problems, all become progressively impacted in both areas. Before diagnosis, individuals living with LOPD often have an abnormal gait and trip easily. Some people living with LOPD require nocturnal noninvasive ventilation if they experience morning headaches or sleep apnea. Others also use it in the day when tired or lying down. Nocturnal noninvasive ventilation use can disturb people’s sleep due to noise or ill-fitting masks. However, individuals living with LOPD who do not use nocturnal noninvasive ventilation may need to sleep upright, which is also disruptive. Invasive ventilation is uncommon in people living with LOPD. Living with constant ventilation (invasive or noninvasive) requires planning because the ventilator needs to be transported with the individual and needs a power source.

Individuals living with LOPD reportedly often experience progressive muscle pain, especially in their lower backs due to weakness of muscles used for walking and/or sitting. Thus, lower back pain persists even with use of a wheelchair. One HCP suggested that all individuals living with LOPD experience fatigue but that the extent is variable between individuals. Swallowing can be a problem for individuals living with LOPD, and eating can become a chore as they need to eat carefully due to risk of aspiration or choking and need to breathe between eating. Some people living with LOPD require a special diet.

The HCPs reported that individuals living with LOPD strive for independence but find household tasks more effortful and often depend on others. Individuals living with LOPD are unable to or have difficulty standing from sitting. This can impact them in their daily activities and socially, and they may avoid going out to eat as they are unsure of their ability to stand up from unfamiliar toilets.

### Vignette Validation and Adaptation

Individuals living with LOPD and HCPs provided feedback on draft health state vignettes, which were iteratively revised following the interviews. All health states, apart from the invasive ventilatory support state, which was only reviewed by HCPs, were reviewed or informed by an interview with someone with LOPD either currently in the health state or who had previous experience with it. Individuals living with LOPD and HCPs found the majority of statements accurate, although some severities of impacts were adjusted following the interviews.

Following feedback from individuals living with LOPD, new statements were added to the health states describing dependence on others; bladder control problems; and frustration. A statement on balance problems and fear of falling was also added to health states describing individuals living with LOPD with either no or intermittent mobility support. The HCPs highlighted difficulties with swallowing and potential for choking in progressed states of individuals living with LOPD using intermittent ventilatory support, which was added to the health state descriptions. The HCPs also provided supportive evidence for the inclusion of statements on balance and falling, frustration, and difficulties with bladder control in the final vignettes.

Table 2 shows details of changes made to the statements included in the health state vignettes following review by HCPs and individuals living with LOPD.

**Table 2.** Interview Feedback from Individuals Living With LOPD and HCPs Informing Changes Made to Health State Vignette Dimensions

**Dimension**	**Feedback (Reviewer)**	**Modification**
Assistance	**All HS:** Dependence is a significant HRQoL impact (individual living with LOPD/HCP) **HS2:** Need help with something most days (individual living with LOPD) **HS4:** Could not live independently (individual living with LOPD). **HS5:** Depend on others for self-care (HCP)	A statement on dependence on others for help was added to all health states Frequency was varied by health state following feedback from individuals living with LOPD
Walking and stairs	**HS3:** Less severe walking problems (individual living with LOPD) **HS4:** Conflicting feedback (individuals living with LOPD)	Severity of walking problems reduced in HS3 and HS4 from trial data draft using feedback from individuals living with LOPD
Balance	**HS2**: Balance gradually gets worse (HCP), and a patient often fell, so avoided walking (individual living with LOPD) **HS4**: Falling biggest HRQoL impact currently (individual living with LOPD)	A statement on balance problems and fear of falling was added to all health states except wheelchair-dependent ones (HS5, HS6, HS7) Severity varied by health state
Reaching	**HS3**: Mixed ability (individual living with LOPD) **HS5**: Very difficult (HCP) **HS6** (individual living with LOPD) and **HS7** (HCP): Unable to reach above their head	Severity increased to “unable to” following feedback from someone living with LOPD (HS6) and HCPs (HS7)
Self-care	**HS3**: Some or mild difficulties (individual living with LOPD) **HS5 and HS6**: Severe difficulties with self-care and need assistance (individual living with LOPD /HCP)	Severity of self-care impact reduced in HS3 and increased in HS5 and HS6
Showering	**HS5**: Need to be transferred from wheelchair to shower (individual living with LOPD and HCP)	Ability to shower separated from main self-care statement to highlight differences between health states Inclusion of detail about needing a wheelchair adapted bathroom and hoist in wheelchair-dependent health states
Bladder control	**HS2**: Mixed feedback (individual living with LOPD) **HS4**: Have bladder control problems (individual living with LOPD/ HCP)	A statement on bladder control problems added to all health states Severity varied by health state
Eating	**HS2**: Occasional difficulties preparing a meal (individual living with LOPD) **HS5**: Conflict between able (HCP) and unable to prepare a meal (individual living with LOPD). **HS4 and HS6**: Difficulties eating, sensation of choking, some difficulties swallowing (individual living with LOPD/HCP) **HS7**: Swallowing difficulties and eating becomes a chore (HCP)	In HS2 and HS5, difficulties preparing a meal were added In HS4 and HS6, difficulties eating were added
Usual activities	**HS1**: Slight difficulties but typically still in full-time employment (HCP) **HS2**: Moderate difficulties, couldn’t pick up dropped things (individual living with LOPD) **HS3**: Wide range of impact severity (individual living with LOPD) **HS5**: Can work/study but not do chores (individual living with LOPD)	Severity decreased from unable to do chores to able but with moderate difficulties in HS2, HS3, HS4 In HS5, overall statement severity reduced but inability to do chores maintained
Pain and discomfort	**HS1**: Slight to moderate pain (individuals living with LOPD /HCP) **HS3**: Range of impact severity (individuals living with LOPD)	Severity was reduced in HS3 and HS4
Frustration, anxiety, and depression	**All HS**: Depression linked to chronic nature of condition and frustration common (HCP) **HS2**: Very frustrated (individuals living with LOPD) **HS3**: Not generally anxious just about, eg, going out (individual living with LOPD) **HS5**: Anxiety, depression and frustration reported (individual living with LOPD)	Frustration added to all health states following feedback from an HCP and individuals living with LOPD Severity of anxiety and depression was reduced in HS3 and increased in HS5
Sleep and fatigue	**HS1**: Mild fatigue (HCP) **HS2**: Nearly always fatigued, sometimes sleeps during day (individual living with LOPD) **HS3, HS4, and HS6**: Sleep disturbance depending on ventilator adjustment (individual living with LOPD/HCP)	Frequency of fatigue reduced in HS1 Sleep disturbance added to intermittent ventilatory support health states (HS3, HS4, HS6)
Socializing	**HS1**: Mild physical limitation socializing only (HCP) **HS3 and HS4**: Need to plan (individual living with LOPD) **HS6**: Feel different from others and difficulty socializing (individuals living with LOPD)	Problems socializing removed from HS1 Some difficulties added to HS3 and HS4
Sport	**All HS**: Different personal definitions of “sport” when evaluating ability (individual living with LOPD)	Feedback from individuals living with LOPD on ability to do sport not comparable, so the statement was removed from all health states

Abbreviations: HCP, healthcare professional; HRQoL, health-related quality of life; HS, health state; LOPD, late-onset Pompe disease.

“Description” and “standing from seated” health state dimensions were not altered following feedback from HCPs and individuals living with LOPD.

### Health State Utility Valuation

Table 3 presents the demographic characteristics and EQ-5D-5L scores of the participants who took part in the health state valuation interviews, alongside representative values for the UK population. Participants were on average 42.9 (SD = 17.7) years old and 49% female. The sample was broadly representative of the UK population but slightly older and with better quality of life.

**Table 3.** Sociodemographic Characteristics of UK TTO Interview Participants

**Characteristic**	**Participants (n = 100)**	**UK Population**
Age		
Mean (SD)	42.9 (17.7)	—
Range	18.0, 85.0	—
Median	42.0	39.4^a^
Sex		
Male	51 (51%)	49%^a^
Female	49 (49%)	51%^a^
Ethnicity		
White	80 (80%)	86%^a^
Mixed or multiple ethnicity	7 (7%)	2%^a^
Asian or Asian British	8 (8%)	8%^a^
Black, African, Caribbean, Black British	4 (4%)	3%^a^
Other ethnic group	1 (1%)	1%^a^
Occupation		
Employed full-time	43 (43%)	—
Employed part-time	19 (19%)	—
Self-employed	8 (8%)	—
Stay at home or full-time carer	1 (1%)	—
Retired	16 (16%)	—
Seeking work/unemployed	2 (2%)	—
Long-term sick leave	0 (0%)	—
Student	11 (11%)	—
Has long-term condition		
Yes	17 (17%)	36%^b^
No	83 (83%)	—
Severity of long-term illness		
Mild	7 (47%)	—
Moderate	8 (53%)	—
Severe	0 (0%)	—
EQ-5D-5L index		
Mean (SD)	0.90 (0.11)	0.856^c^
Range	0.51, 0.99	—
EQ-5D-5L VAS		
Mean (SD)	88.7 (9.6)	82.8^c^
Range	50, 100	—

Abbreviation: VAS, visual analog scale.

^a^Figures based on data from the 2011 UK national census.^22^

^b^Figure based on data from the 2013 UK Opinions and Lifestyle Survey.^38^

^c^Figures based on Janssen et al.^39^

**Figure 2.**
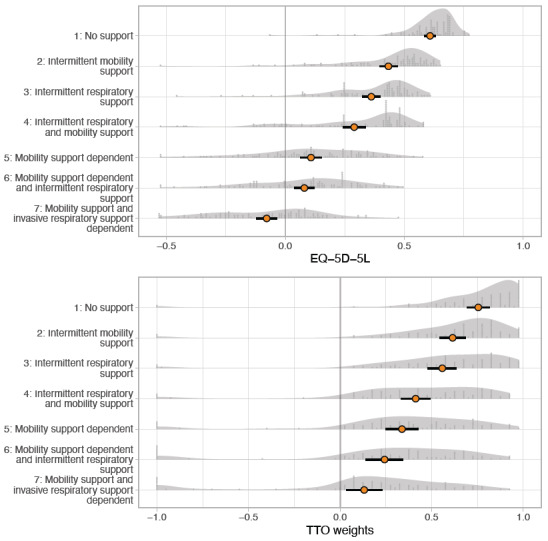
EQ-5D-5L Index Scores and TTO Alongside Density Distribution Abbreviation: TTO, time trade-off. Mean (95% CI) EQ-5D-5L index scores (top) and TTO weights (bottom); (n = 100).

Results of the valuation exercises of each health state are presented in Table 4. The distributions of the TTO and EQ-5D-5L utilities are shown in Figure 2. Participants rated health states progressively worse with increased dependency on assistive technology in all 3 exercises. The mean VAS scores ranged from 63.4 (no support) to 26.7 (invasive ventilatory and mobility support-dependent). Mean TTO and EQ-5D-5L utilities followed a similar pattern and ranged from 0.754 to 0.132 and 0.608 to -0.078, from no support to invasive ventilatory and mobility support–dependent, respectively. In the TTO exercise, participants used the complete range of possible utilities (-1 to 1) in all health states. EQ-5D-5L utility values worse than dead (<0) were obtained in all health states, in particular in the invasive ventilatory and mobility support–dependent state.

**Table 4.** Utility Valuations of Health States: VAS Scores, TTO Weights, <br>EQ-5D-5L Index Scores (n = 100)

**Health State**		**VAS**		**TTO Weights**		**EQ-5D-5L Index**	
		**Mean (SD)**	**95% CI**	**Mean (SD)**	**95% CI**	**Mean (SD)**	**95% CI**
1	No support	63.4 (16.5)	60.2, 66.7	0.754 (0.312)	0.693, 0.815	0.608 (0.120)	0.585, 0.632
2	Intermittent mobility support	51.2 (16.8)	47.9, 54.5	0.614 (0.356)	0.544, 0.684	0.433 (0.195)	0.395, 0.471
3	Intermittent ventilatory support	44.9 (17.7)	41.4, 48.4	0.558 (0.398)	0.480, 0.636	0.361 (0.190)	0.324, 0.398
4	Intermittent ventilatory and mobility support	38.7 (17.6)	35.2, 42.1	0.412 (0.413)	0.331, 0.493	0.289 (0.244)	0.242, 0.337
5	Mobility support–dependent	34.8 (17.1)	31.5, 38.2	0.338 (0.448)	0.250, 0.426	0.108 (0.230)	0.063, 0.153
6	Mobility support–dependent and intermittent ventilatory support	32.7 (17.2)	29.3, 36.1	0.243 (0.518)	0.141, 0.344	0.080 (0.220)	0.037, 0.123
7	Mobility support– and invasive ventilatory support–dependent	26.7 (17.0)	23.4, 30.0	0.132 (0.497)	0.034, 0.229	-0.078 (0.221)	-0.122, -0.035

Abbreviations: CI, confidence interval; TTO, time-trade-off; VAS, visual analog scale.

VAS scores were rescaled fixing the dead state at 0. Published self-reported mean (SD) EQ-5D-3L index scores for adult Pompe patients: with mobility support, 0.67 (0.21); with ventilatory support, 0.61 (0.26).

## DISCUSSION

This study was designed to estimate utility data describing different states of LOPD, which were defined by the dependence on mobility and/or ventilatory support. Health state vignettes were developed based on clinical trial PRO data, a literature review, and further refined following interviews with HCPs and individuals living with LOPD. This approach is in line with current best practice guidance^23,40^ and ensured that all important dimensions of HRQoL are accurately described.

The health state valuation results capture the progressive nature of the disease and highlighted the decline in HRQoL with increased dependence on assistive technology, which was consistently observed in the VAS, TTO and EQ-5D-5L utilities. Published utility estimates in Pompe disease are similar to our results and ranged between 0.670^41^ to 0.853^42^ for the mildest (nonsupport) state in adults. Consistent with our findings, another study of adults with LOPD suggested the increased impact on HRQoL when using assistive technology.^43^ Although some utility estimates for Pompe disease existed, their adequacy for use in economic evaluation remained unclear because the methods and definition of health states were poorly reported.^20^ The present study aimed to add to the evidence base by providing robust utility estimates for a wide range of symptomatic individuals living with LOPD, obtained from health state vignettes developed with best available data from multiple sources. The presented utility values can be used to support economic evaluations of novel and existing treatments for LOPD. Across health states, TTO utilities were consistently higher than EQ-5D-5L utilities. The authors assume this is because in the TTO exercise, participants have to trade life-years, which some participants may be more hesitant to do resulting in higher TTO utility estimates. When rating the EQ-5D, participants are oblivious to the associated utilities and to values worse than dead. Rating vignettes using the EQ-5D has been advocated in the Case for Change document released by NICE as part of their methods review.^40^ More work is required to determine how EQ-5D–based assessments of vignettes are consistent with the ratings that individuals living with LOPD in those health states would have provided if they had completed EQ-5D. By presenting both TTO scores and EQ-5D values for the same states, modelers and decision makers can decide which they believe are most appropriate.

EQ-5D-5L utility data obtained in clinical trials may be used for economic modeling, although, as it was in our case, this can be challenging as trials typically exclude individuals in more progressed or severe health states. Using health state vignettes is then a common approach to estimate utilities in rare diseases, but the method has its limitations. The vignettes describe the experience of a “typical” individual living with LOPD in each health state, which does not capture the full variability between individual in the same state that transpired in this study’s qualitative interviews. For example, one individual using intermittent ventilatory and mobility support was able to walk short distances with only minor difficulties, whereas others with the same level of assistive technology use reported severe walking problems. The vignettes are a simplification of what it is like to live in this state, and an individual’s experience will be personal. This study focused on individuals living with LOPD who are symptomatic and eligible for ERT treatment, thus the utility values, particularly for the nonsupport state, may be lower than in the general LOPD population who use no support.

It is challenging to establish the validity of the vignette content. By using PRO clinical trial data for the initial vignette draft, the descriptions of the main HRQoL dimensions were based on quantitative evidence. The vignettes were then supplemented by literature evidence and in-depth qualitative interviews with HCPs and individuals living with LOPD. We believe that by using both quantitative trial data and qualitative interviews, the vignettes were mostly accurate descriptions of the experiences of people living with LOPD. Using multiple data sources is required, as all have their own limitations. Trial data were limited to a few health states as the clinical trial did not include individuals living with LOPD in severe (mobility support–dependent, invasive ventilatory support–dependent) states. The PROs included in the clinical trial (EQ-5D-5L and R-PAct) did not cover all HRQoL dimensions and important areas (ie, sleep/fatigue, balance problems, urinary issues, frustration, social impact, and dependence on others) were missed. In interviews with individuals living with LOPD, participants often described being able to do certain activities but requiring accommodations or assistance to do so. As the individuals interviewed have been living with LOPD for some time, they may have become accustomed to their limitations and may be less able to identify and report difficulties in interviews. Additionally, the individuals living with LOPD interviewed were only presented with health states they are currently experiencing or have experienced and not health states they may experience in the future due to concerns about their emotional well-being. Thus, it was not possible to obtain feedback from individuals living with LOPD on all health states, and the last health state was reviewed by HCPs only.

A further limitation of this study type is that the health state valuation exercise can be difficult for members of the general public. It is difficult to imagine the impact of being mobility support–dependent and to comprehend the disease burden of LOPD. Especially for more severe health states, the public may find it harder to imagine being in the health state, which could explain the higher uncertainty around later stages compared with earlier states.

## CONCLUSION

This study provides a set of robust utility values for HRQoL in LOPD that can be used in economic modeling of new or existing treatments in LOPD and demonstrate the value UK society places on delaying disease progression. The obtained utility values align well with reported utility values for earlier stages of Pompe disease and add new utility values for more progressed states.^43^ The results are based on robust evidence for the health state descriptions, obtained by clinical trial data review and extensive interviews with HCPs and individuals living with LOPD.

### Author Contributions

L.H., A-K.S., A.M., R.G., S.R., A.J.L., and A.S. contributed to the conception and design of the study. L.H., A-K.S., D.H., L.M., and K.G. contributed to the acquisition of data. L.H., A-K.S., and K.G. contributed to the analysis of the data and drafted the manuscript. All authors contributed to the interpretation of the data, critical revision, and approval of the final manuscript.

### Disclosures

A.M., R.G., S.R., and A.S. are employed by and hold stock in Amicus Therapeutics. L.H., A-K.S., and K.G. are employees of Acaster Lloyd Consulting Ltd. A.J.L. is an employee and shareholder of Acaster Lloyd Consulting Ltd. Acaster Lloyd Consulting Ltd was commissioned by Amicus Therapeutics to conduct the study. D.H. and L.M. have received consulting fees from Amicus Therapeutics not in relation to this project. D.H. has received honoraria for speaking and advisory boards from Amicus Therapeutics and Sanofi administered through UCL consultants and used in part for lysosomal storage disorder–related research.

## Supplementary Material

Online Supplementary Material
