# Supplementary material for: Quality of Life with Late-Onset Pompe Disease: Qualitative Interviews and General Public Utility Estimation in the United Kingdom
Source: J Health Econ Outcomes Res. 2023 Mar 3;10(1):41–50. doi: 10.36469/001c.68157 (PMC9985911; doi:10.36469/001c.68157)
Supplement: Online Supplementary Material [file jheor_2023_10_1_68157_151135.pdf]

Journal of Health Economics and Outcomes Research

Online Supplementary Material

Quality of Life With Late-Onset Pompe Disease: Qualitative Interviews and General Public Utility Estimation in the United Kingdom. *JHEOR*. 2023;10(1):41-50. doi:10.36469/jheor.2023.68157

Appendix 1: Health State Vignettes

Appendix 2: Results of Trial Data Review

This supplementary material has been provided by the authors to give readers additional information about their work.

This is an open-access article distributed under the terms of the Creative Commons Attribution 4.0 International License (CCBY-4.0). View this license's legal deed at http://creativecommons.org/licenses/by/4.0 and legal code at http://creativecommons.org/licenses/by/4.0/legalcode for more information.

APPENDIX 1: HEALTH STATE VIGNETTES

1: No support

- You have a rare, genetic disease that causes progressive muscle weakness.
- You depend on others for help occasionally.
- You are able to stand up from a seated position, but with difficulties.
- You have slight to moderate walking problems and are unable to run. You are able to walk short distances and up and down stairs, but with difficulties.
- You have no balance problems and are not scared of falling.
- You are able to reach for an object and grasp above your head without difficulties.
- You have no problems with self-care and can comb your hair and get dressed without difficulties.
- You can shower without difficulties.
- You have no problems with bladder control.
- You are able to prepare a meal and eat without difficulties.
- You have slight problems with usual activities such as work, study, household chores, and leisure activities.
- You have slight to moderate pain or discomfort.
- You are sometimes frustrated, anxious or depressed.
- You sometimes have disturbed sleep. You sometimes feel fatigued during the day.
- You are able to socialize with your friends and family.

2: Intermittent mobility support

- You have a rare, genetic disease that causes progressive muscle weakness. You use a mobility support, such as a walking aid, wheelchair or motorized scooter some of the time.
- You sometimes depend on others for help.
- You are able to stand up from a seated position, but with difficulties.
- You have moderate walking problems and are unable to run. You are able to walk short distances and up and down stairs, but with difficulties.
- You have moderate balance problems and are often scared of falling.
- You are able to reach for an object and grasp above your head without difficulties.
- You have slight problems with self-care but can comb your hair without difficulties. You can get dressed, but with difficulties.
- You can shower without difficulties.
- You have no problems with bladder control.
- You have occasional difficulties preparing a meal but can eat without difficulties.
- You have moderate problems with usual activities such as work, study, household chores, and leisure activities.
- You have slight to moderate pain or discomfort.
- You are often frustrated and occasionally anxious or depressed.
- You sometimes have disturbed sleep. You often feel fatigued during the day.
- You have some difficulties socializing with your friends and family.

3: Intermittent respiratory support

- You have a rare, genetic disease that causes progressive muscle weakness. You need a machine to help you breathe during the night or sometimes during the day.
- You sometimes depend on others for help.
- You are able to stand up from a seated position, but with difficulties.
- You have moderate walking problems and are unable to run. You are able to walk short distances and up and down stairs, but with difficulties.
- You have no balance problems and are not scared of falling.
- You are able to reach for an object and grasp above your head, but with difficulties.
- You have slight to moderate problems with self-care. You can comb your hair without difficulties and can get dressed, but with difficulties.
- You can shower, but with difficulties.
- You have some problems with bladder control.
- You have difficulties preparing a meal but can eat without difficulties.
- You have moderate problems with usual activities such as work, study, household chores, and leisure activities.
- You have moderate pain or discomfort.
- You are often frustrated and occasionally anxious or depressed.
- You have slightly disturbed sleep. You occasionally feel fatigued during the day.
- You are able to socialize with your friends and family.

4: Intermittent respiratory and mobility support

- You have a rare, genetic disease that causes progressive muscle weakness. You use a mobility support, such as a walking aid, wheelchair or motorized scooter some of the time and need a machine to help you breathe during the night or sometimes during the day.
- You often depend on others for help.
- You are able to stand up from a seated position, but with difficulties.
- You have moderate walking problems and are unable to run. You are able to walk short distances, but with difficulties. You have severe difficulties walking up and down stairs.
- You have moderate balance problems and are often scared of falling.
- You are able to reach for an object and grasp above your head, but with difficulties.
- You have moderate problems with self-care. You can comb your hair without difficulties and can get dressed, but with difficulties.
- You can shower, but with difficulties.
- You have some problems with bladder control.
- You have difficulties preparing a meal and can eat, but with occasional difficulties.
- You have moderate problems with usual activities such as work, study, and leisure activities. You are able to do limited household chores.
- You have moderate pain or discomfort.
- You are often frustrated and occasionally anxious or depressed.
- You have slightly disturbed sleep. You sometimes feel fatigued during the day.
- You have some difficulties socializing with your friends and family.

5: Mobility support–dependent

- You have a rare, genetic disease that causes progressive muscle weakness. You use a wheelchair or motorized scooter all of the time.
- You almost always depend on others for help.
- You are unable to stand up from a seated position.
- You are unable to walk and are able to get around using your wheelchair.
- You are able to reach for an object and grasp above your head, but with difficulties.
- You have severe problems with self-care. You can comb your hair and get dressed, but with difficulties.
- You can shower and use the toilet in a wheelchair-adapted bathroom and may require a hoist to assist.
- You have some problems with bladder control.
- You have difficulties preparing a meal but can eat without difficulties.
- You have moderate problems with usual activities such as work, study, and leisure activities. You are unable to do household chores.
- You have slight to moderate pain or discomfort.
- You are often frustrated and occasionally anxious or depressed.
- You sometimes have disturbed sleep. You often feel fatigued during the day.
- You have some difficulties socializing with your friends and family.

6: Mobility support–dependent and intermittent respiratory support

- You have a rare, genetic disease that causes progressive muscle weakness. You use a wheelchair or motorized scooter all of the time and need a machine to help you breathe during the night or sometimes during the day.
- You almost always depend on others for help.
- You are unable to stand up from a seated position.
- You are unable to walk and are able to get around using your wheelchair.
- You are unable to reach for an object and grasp above your head.
- You have severe problems with self-care. You can comb your hair and can get dressed, but with difficulties.
- You can shower and use the toilet in a wheelchair-adapted bathroom and may require a hoist to assist.
- You have some problems with bladder control.
- You have difficulties preparing a meal and can eat, but with difficulties.
- You have moderate problems with usual activities such as work, study, and leisure activities. You are unable to do household chores.
- You have moderate pain or discomfort.
- You are often frustrated and sometimes anxious and depressed.
- You have slightly disturbed sleep. You rarely feel fatigued during the day.
- You have some difficulties socializing with your friends and family.

7: Mobility support– and invasive respiratory support–dependent

- You have a rare, genetic disease that causes progressive muscle weakness. You use a wheelchair or motorized scooter all of the time and always need a machine to help you breathe via a tube inserted in your windpipe.
- You almost always depend on others for help.
- You are unable to stand up from a seated position.
- You are unable to walk and are able to get around using your wheelchair.
- You are unable to reach for an object and grasp above your head.
- You have severe problems with self-care. You can comb your hair and get dressed, but with difficulties.
- You can shower and use the toilet in a wheelchair-adapted bathroom and may require a hoist to assist.
- You have some problems with bladder control.
- You have difficulties preparing a meal and eating.
- You are unable to do usual activities such as work, study, household chores, and leisure activities.
- You have moderate pain or discomfort.
- You are often frustrated and sometimes anxious and depressed.
- You have slightly disturbed sleep. You often feel fatigued during the day.
- You have difficulties socializing with your friends and family.

APPENDIX 2: RESULTS OF TRIAL DATA REVIEW

Table 1. Item-Level Response (Median) of Clinical Trial Participants on the EQ-5D-5L and R-PAct

Characteristic	HS1: No Support	HS2: Intermittent Mobility Support	HS3: Intermittent Respiratory Support	HS4: Intermittent Respiratory and Mobility Support
**EQ-5D-5L**	n = 66	n = 16	n = 4	n = 1
Mobility	2.0	3.0	4.0	3.0
Self-care	1.0	2.0	3.0	2.0
Usual activities	2.0	3.0	3.0	3.0
Pain/ discomfort	2.0	2.5	3.5	3.0
Anxiety/ depression	1.0	1.0	3.5	2.0
**R-PAct**	n = 52	n = 15	n = 3	n = 1
Comb hair	2.0	2.0	2.0	2.0
Eat	2.0	2.0	2.0	2.0
Put on trousers	2.0	1.0	1.0	2.0
Prepare meal	2.0	2.0	1.0	2.0
Take shower	2.0	2.0	1.0	2.0
Reach for an object above the head	2.0	2.0	1.0	2.0
Step over obstacles	1.0	1.0	1.0	2.0
Turn over in bed	1.0	1.0	1.0	2.0
Walk on uneven surfaces	1.0	1.0	1.0	2.0
Stand up from a sitting position	1.0	1.0	1.0	1.0
Walk more than 1 km	1.0	1.0	0.0	1.0
Walk up and down stairs	1.0	1.0	1.0	0.0
Bend over and stand up	1.0	0.0	1.0	0.0
Walk at rapid speed	1.0	0.0	0.0	0.0
Carry out tasks in yard	1.0	0.0	0.0	0.0
Practice a sport	0.0	0.0	0.0	0.0
Squat and stand up	0.0	0.0	1.0	0.0
Run	0.0	0.0	0.0	0.0

Abbreviation: R-PAct, Rasch-built Pompe-specific activity scale.

Intermittent mobility support was defined as use of one or two canes, walking stick, walker, crutches.

Intermittent respiratory support was defined as nocturnal ventilation or use of ventilation in supine position (eg, BI-PAP or CPAP).

EQ-5D-5L: 1, no problems (green); 2, slight problems (yellow); 3, moderate problems (yellow); 4, severe problems (red); 5, extreme problems (red).

R-Pact, 2, able to (green); 1, able to with difficulties (yellow); 0, unable to (red).
